# Faulty Feeder Identification Based on Data Analysis and Similarity Comparison for Flexible Grounding System in Electric Distribution Networks

**DOI:** 10.3390/s21010154

**Published:** 2020-12-29

**Authors:** Kangli Liu, Sen Zhang, Baorun Li, Chi Zhang, Biyang Liu, Hao Jin, Jianfeng Zhao

**Affiliations:** 1 School of Electrical Engineering, Southeast University, Nanjing 210096, China; sen_zhang@seu.edu.cn (S.Z.); 220172670@seu.edu.cn (B.L.); 220192759@seu.edu.cn (C.Z.); 230179573@seu.edu.cn (B.L.); 230169162@seu.edu.cn (H.J.); jianfeng_zhao@seu.edu.cn (J.Z.); 2 Jiangsu Provincial Key Laboratory of Smart Grid Technology and Equipment, Nanjing 210096, China; 3 State Grid Suqian Power Supply Company, Suqian 223800, China

**Keywords:** faulty feeder identification, data analysis, similarity comparison, flexible grounding system, grey T-type correlation degree, wavelet packet transform, electric distribution networks

## Abstract

Reliability and safety are the most important indicators in the electric system. When a ground fault occurs, the electrical equipment and personnel will be greatly threatened. Due to the zero-sequence voltage/current sensor networks applied in the system, the fault identification and diagnosis technology are developing rapidly, including the application of ground fault suppression. A flexible grounding system (FGS) is a new technology applied to arc extinguishing in medium and high voltage electric distribution networks. Its characteristic is that when the single-phase ground fault occurs, the power-electronic-based device is put into the electric system to compensate and suppress the ground point current to be close to zero in a very short time. In order to implement the above process, the corresponding faulty feeder identification method needs to meet the requirements of rapidity and accuracy. In this article, based on the real-time sampled data from the zero-sequence current/voltage sensors, an improved faulty feeder identification method combining wavelet packet transform (WPT) and grey T-type correlation degree is proposed, which features both accuracy and rapidity. The former is used to reconstruct the transient characteristic signal, and the latter is responsible for calculating and comparing the similarity of relative variation trend. Simulation results verify the rationality and effectiveness of the proposed method and analysis.

## 1. Introduction

**Figure 1:**
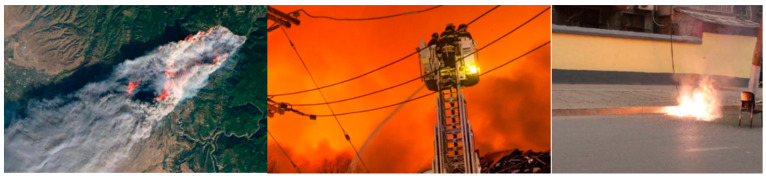
Fire potentially caused by the ground fault in electric distribution networks.

As an important part of the electric system, the medium and high voltage electric distribution networks are closely related to all industrial and residential users. Its safety and reliability have always been a research hotspot that has been widely discussed. Among many fault types of distribution networks, the single-phase ground fault is more frequent in practice. When a ground fault occurs in the system, the safety of electrical equipment and personnel will be greatly threatened. For example, fires potentially caused by ground faults have occurred in many countries, as shown in Figure 1. Therefore, the implementation of reliable and effective grounding is an important premise to ensure the safety of distribution networks. In recent years, with the continuous development of the distribution network topology and the increasing number of cable lines, the resonant grounding method (arc suppression coil (ASC) or Peterson coil) has been difficult to meet the requirement of rapid arc extinction. In order to solve this problem, the power-electronics-devices-based flexible grounding system (FGS) has been proposed in recent years [1,2,3,4,5,6]. Flexible grounding means that on the basis of traditional resonant grounding, when a single-phase ground fault occurs, power-electronic-based devices will be put into the distribution network, and the fault current at the ground point is suppressed to be close to zero in a very short period of time. In addition, the fault arc will be completely extinguished. The existing flexible grounding schemes include open-loop and closed-loop compensation schemes [7,8]. For the former, it is estimated to obtain the compensation current, and the active inverter is equivalent to the current source. The working principle of the inverter in this scheme is similar to an active power filter (APF). For the latter, it feeds back the voltage at the fault point, and the active inverter is equivalent to the voltage source. The output voltage forces the fault point voltage to be equal to zero or below the safety threshold. However, no matter which kind of flexible grounding scheme is adopted, higher requirements are put forward for the faulty feeder selection technology: (1) Accuracy: The faulty feeder identification results determine the parameter values calculation in the compensation process, which will directly affect the reliability of FGS. Especially, when a high-resistance ground fault occurs, smaller fault characteristics will make the feeder identification process more difficult; (2) rapidity: FGS needs to realize arc extinguishing in a very short time (usually several power frequency cycles), which makes the feeder identification process need to be completed in a very short time. In addition to the faulty feeder identification, fault detection and location are also very important in the electric system, which are not the focus of this paper. Some effective methods have been proposed [9,10,11,12,13].

At present, the faulty feeder identification methods for the grounding system can be roughly divided into four categories [14,15,16,17,18,19,20,21,22,23], and they are all based on the transient signals analysis: (1) First half-wave method; (2) energy method; (3) time-frequency domain analysis method; (4) similarity comparison method. The first half-wave method compares the initial polarity of the bus transient zero-sequence voltage of the bus and the polarity of the transient zero-sequence current of each feeder. The line with the opposite polarity of the transient zero-sequence voltage of the bus is the faulty feeder [24,25]. However, this method needs to collect the waveform of the first half period after the fault, which requires high speed and data synchronization, and its reliability is poor. The energy method uses the transient zero-sequence voltage and current of the non-faulty/faulty feeder first to obtain the energy of the transient process and then realizes the fault line selection by comparing the difference in energy magnitude and direction [26]. However, this method ignores large amounts of high-frequency signals, which can easily lead to false selection. The time-frequency domain analysis method mainly includes the wavelet transform (WT) and wavelet packet transform (WPT) [27]. In addition, there are some other time-frequency domain analysis methods that are utilized to extract the transient feature, such as the Fourier transform (The time-domain signal is transformed into the frequency-domain signal) [28], short-time Fourier transform (STFT, and the window function is added) [29], empirical mode decomposition (EMD) [30], Hilbert–Huang Transform (HHT) [31,32], Wigner–Ville distribution (WVD) [33], and so on. WPT improves high-frequency resolution on the basis of WT, thus it is more conducive to fault signal processing [34,35,36]. It first decomposes the fault transient signal in each frequency band, then determines the frequency band that can reflect the fault characteristics most obviously, and finally uses the amplitude, polarity and phase, entropy value, and other characteristic quantities of the signal in the band to select the fault line. The WPT method has strong signal extraction ability and can realize the transient signals processing in a short time. However, the accuracy of the method is affected by the sampling frequency and the complexity of the distribution network. Compared with the Fourier transform, the signal with WPT can be processed within one or two fundamental current cycles, which means the signal processing has a better real-time performance. Besides that, WPT is more suitable for time domain and frequency domain analysis at the same time. In addition, for the Fourier transform, as the basis functions are sine or cosine functions, it is more suitable for the analysis of stationary signals. To solve this, the window function is added to the Fourier transform (STFT). However, the selection of the window function will seriously affect the time-frequency resolution of signal analysis. The other methods, such as EMD, HHT, and Wigner–Ville distribution, are more widely used in mechanical fault diagnosis, power quality assessment, and other fields. The similarity comparison method determines the similarity between the zero-sequence current waveforms of the feeder by finding the correlation coefficient or grey correlation degree and finds the feeder with the most different from other lines as the fault feeder [37,38,39,40,41,42,43]. This method has higher line selection accuracy than other methods mentioned above but requires a longer period of the waveform to extract the transient components contained in the fault waveform. In [40], in order to extract the transient characteristic quantity, the zero-sequence current waveform after 10 cycles is subtracted from the zero-sequence current when the fault occurs. Hence, its dynamic performance will be affected. In summary, none of the above four methods can meet the requirements of accuracy and rapidity at the same time, and, therefore, it is not suitable for a flexible grounding scheme. An improved faulty feeder identification method for FGS is required.

**Figure 2:**
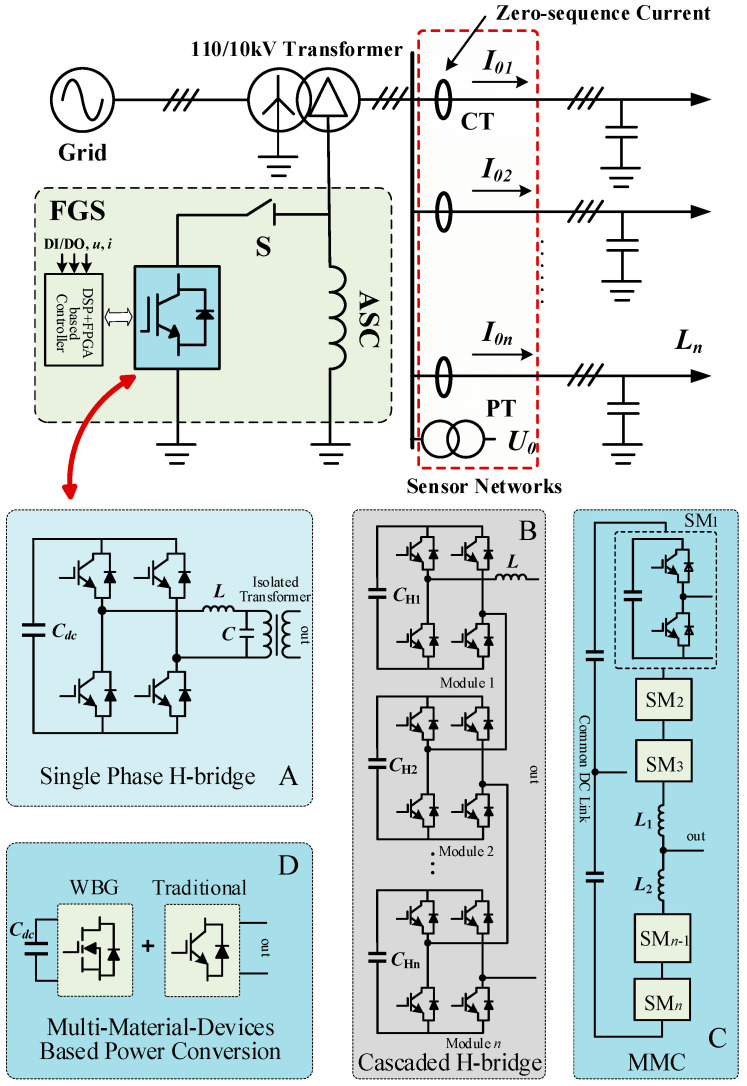
Schematic diagram of the flexible grounding system (FGS)-based electric distribution network with zero-sequence current/voltage sensors. And the topologies of the active inverter can be: (**A**) single-phase H-bridge; (**B**) cascaded H-bridge; (**C**) modular multilevel converter (MMC); (**D**) multi-material devices-based topology.

**Figure 3:**
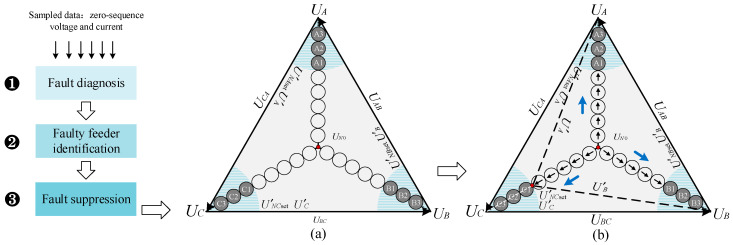
Schematic diagram of neutral-point voltage compensation strategy. (**a**) Before the compensation; (**b**) after the compensation.

Due to the large number of zero-sequence voltage and current sensors in the electric distribution network, the accurate and rapid fault identification result can be achieved. In addition, the key to faulty feeder identification is to separate fault information from a large number of real-time sampled data. Figure 2 and Figure 3 show the schematic diagrams of FGS and its neutral-point voltage compensation strategy. The hardware mainly includes an active inverter, impedance grounding system (ASC or low-resistance), and sensor networks. When a ground fault occurs, the fault-point voltage exceeds the safety threshold, and the fault arc is thus formed. Since the sum of the fault-phase voltage (*U_A_*, *U_B_*, *U_C_*) and neutral-point voltage (*U_NO_*) is equal to the fault-point voltage, in order to suppress the fault-point voltage and make it below the safety threshold, the active inverter can be used to compensate the neutral-point voltage, which can offset the fault-phase voltage. ${U^{\prime }}_{NA\mathrm{set}}$, ${U^{\prime }}_{NB\mathrm{set}}$ and ${U^{\prime }}_{NC\mathrm{set}}$ are the compensated neutral-point voltages. In Figure 3, the blue areas represent the safe range of the fault-phase voltage value. That is, when the fault-phase voltage is offset to this range, the fault point can not arcing, and the ground fault is effectively suppressed. Additionally, according to different application scenarios, the topology structure of the active inverter can be single-phase H-bridge, cascaded H-bridge, modular multilevel converter (MMC), and multi-material devices [44,45,46,47,48,49,50,51,52,53]. In order to achieve the high voltage output of the active inverter, the multilevel topology or cascaded H-bridge can be adopted [44,45,46,47,48,49]. If the output voltage level of the active inverter is lower, an isolation booster transformer is necessary. In addition, in order to achieve the tradeoff of power density, performance, and cost, multi-material devices can be applied [50,51,52,53]. The whole software process includes fault diagnosis, fault feeder identification (mainly discussed in this article), and suppression. Firstly, the controller of the flexible grounding system samples the signal data of zero-sequence voltage and current transformer, then performs necessary data processing and analysis, and finally sends driving signals to power electronic devices, thus that the voltage or current at fault point will be suppressed to a safe range.

In this article, combining the advantages of wavelet packet transform and the similarity comparison method based on grey T-type correlation degree, a new faulty feeder identification method with both excellent accuracy and speed is proposed. The wavelet packet transform is applied to locate the fault moment and extract the transient characteristic quantity in the fault waveform quickly and accurately. In addition, the grey correlation degree is applied to analyze the similarity of the relative change trend of each feeder waveform to achieve faulty feeder identification. The zero-sequence voltage and current sensor networks provide necessary real-time data support for algorithm implementation. Sufficient simulation results based on MATLAB/Simulink verify the validity and rationality of the proposed method.

This paper is organized as follows. The description of wavelet packet transformation is given in Section 2, including the principle and extraction of zero-sequence current transient characteristics. Additionally, in this section, the processing of transient waveforms using WPT is introduced. In Section 3, the similarity comparison method based on grey T-type correlation degree is presented. Especially, the flow chart of the proposed method combining WPT and grey T-type correlation degree are given. Taking into account several typical ground fault cases, the data analysis and simulation results are presented in Section 4. Finally, Section 5 summarizes the whole work.

## 2. Wavelet Packet Transformation for FGS

### 2.1. Principle of Wavelet Packet Transform

**Figure 4:**
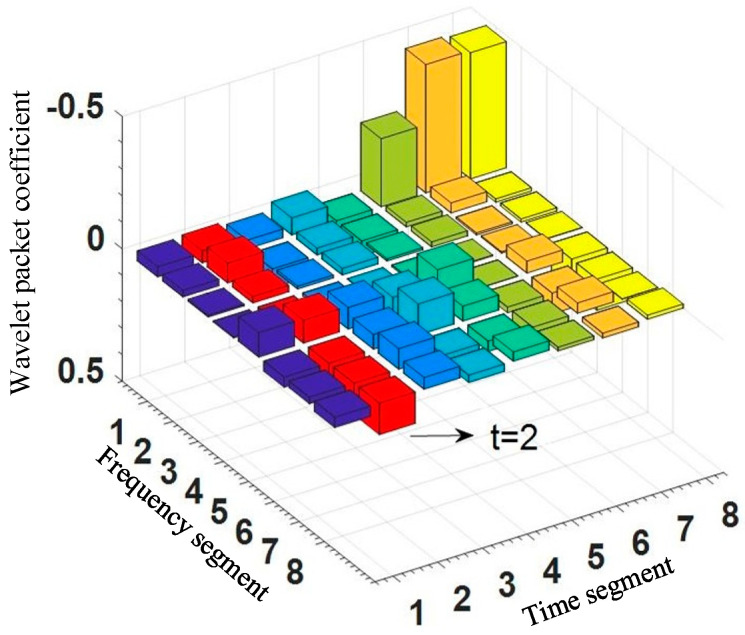
Schematic diagram of the three-layer wavelet packet decomposition example.

Wavelet packet transform is a method of time-frequency domain analysis. Compared with the Fourier transform, the signal with WPT can be processed within one or two fundamental current cycles. Compared with wavelet transform, the frequency band can be divided into multiple layers with WPT, and the high-frequency part of the signal is further decomposed, thus as to ensure that the signal features are complete and has good time-frequency resolution. WPT performs high-frequency and low-frequency filtering on the signal over a period of time. Each transformation separates the low-frequency overview and high-frequency details of the signal. After decomposing *n* layers, according to the above steps, the wavelet packet coefficients in 2^n^ frequency bands are obtained. Figure 4 is a schematic diagram of the three-layer wavelet packet decomposition example. The time and frequency domains are equally divided into eight segments. At a certain period of time, such as *t* = 2, the signal characteristics in eight frequency bands can be represented by wavelet packet coefficients, which reflect the projection value of the signal on each base component. It is related to the wavelet base, signal sampling frequency, and the number of decomposition layers. After the wavelet packet coefficients are obtained, the signal can be reconstructed in each frequency band according to the proportion of each component in the signal to obtain the waveform of the signal in each frequency band.

### 2.2. Extraction of Zero-Sequence Current Transient Characteristic

In the normal operating state, since the flexible compensation device is not put into use, it is the same as the transient equivalent circuit of the single-phase ground fault in the traditional resonant grounding system. The transient current *i_d_* at the ground point [37,40] is:

(1) $$\begin{array}{ll} i_{d}=i_{L}+i_{C} & =(I_{Cm}-I_{Lm})\cos(\omega t+\varphi )+I_{Lm}\cos\varphi e^{-\frac{t}{\tau_{L}}}\\ & \;+I_{Cm}(\frac{\omega_{f}}{\omega }\sin\varphi \sin\omega_{f}t-\cos\varphi \sin\omega_{f}t)e^{-\frac{t}{\tau_{C}}} \end{array}$$

where *I_Cm_* and *I_Lm_* are the amplitude of the transient capacitor current and inductor current; *ω* is the angular frequency of the power grid; *φ* is the initial phase angle of the phase voltage at the time of fault; *ω_f_* is the resonance frequency of the loop; *τ_L_* and *τ_C_* are the time constant of the inductive loop and the capacitive loop.

**Figure 5:**
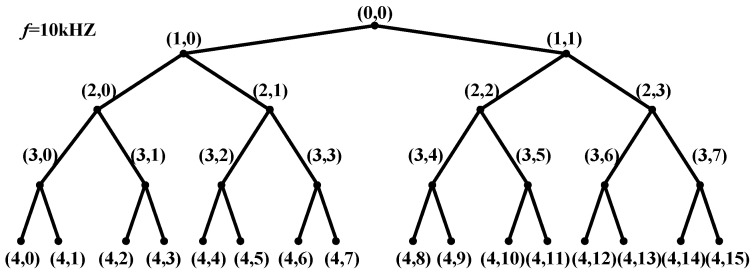
Schematic diagram of four-layer wavelet packet tree.

This article applies the wavelet packet transform to the processing of transient waveforms, which mainly includes two steps:

(1) Determine the actual fault time—the system continuously collects the bus zero-sequence voltage and feeder zero-sequence current during normal operation and saves at least two cycles of data. In order to distinguish between ground fault and voltage unbalance, the zero-sequence voltage threshold *U*_set_ is set to 15% of the maximum phase voltage, thus as to identify 0–2 kΩ ground fault. When the instantaneous value of the zero-sequence voltage exceeds the threshold, it is judged that a fault has occurred. However, sometimes it is affected by the grounding resistance or other conditions, and the instantaneous value of the zero-sequence voltage does not immediately cross the boundary. That is to say, the fault time *t*_1_ obtained according to the above method often lags the actual fault time *t*_0_. In this article, the Coiflet5 wavelet is used to transform the zero-sequence voltage using the wavelet packet singularity principle to determine the maximum point. At this point, the signal mutation is the most obvious, corresponding to the actual fault time *t*_0_.
(2) Transient characteristic waveform extraction—under typical conditions, such as the broken wire-to-ground fault, the ground medium is the cement floor, weeds and so on. The transition resistance is usually hundreds to thousands of ohms. Therefore, when this kind of fault occurs, the fault current is mainly composed of the fundamental current. In addition, due to the existence of nonlinear load, the fault current will also contain some low-frequency harmonic current components. Thus, in the fault current, 1st, 3rd, and 5th signal amplitude are the largest, and their characteristics are most obvious when the fault occurs. Using this as the reference waveform for line selection can reduce the interference of high frequencies and other uncontrollable factors. Select the sampling frequency as 10 kHz, consider the boundary effect, and take the feeder zero-sequence current signals of 1/4 and 3/4 cycles before and after the fault time to perform four-layer wavelet packet decomposition, and the schematic diagram of the four-layer wavelet packet tree is shown in Figure 5. According to Shannon’s sampling theorem, the effective frequency bandwidth represented by each node on the fourth layer = 10,000/2/16 = 312.5 Hz. Therefore, the reconstructed signal at node (4, 0) can accurately extract the signal of 0–312.5 Hz through only one period of sampling signal. The principle of this method is equivalent to low-pass filtering without time delay.

## 3. Feeder Identification Method Based on Grey T-Type Correlation Degree

### 3.1. Characteristic Analysis of Transient Zero-Sequence Current

When the ground fault occurs, the zero-sequence current of non-faulty feeder *p* can be expressed as:

(2) $$i_{op}=C_{p}\frac{du_{0}}{dt},p=1,2,\ldots ,m,p\neq q$$

where *U*_0_ is the system zero-sequence voltage; *C_p_* is the capacitance to ground of the feeder *p*.

It can be found from (2) that *i_op_* is obtained by multiplying the 2 parts, and its value depends on its own capacitance to the ground and the change rate of the zero-sequence voltage. Different feeders have different capacitances to ground, which resulted in different zero-sequence current amplitudes, but different feeder zero-sequence currents contained the same zero-sequence voltage change rate. Therefore, the dynamic change trend was consistent, which provides a theoretical basis for extracting feature quantities and judging the similarity between non-faulty feeders.

The zero-sequence current of the faulty feeder *q* can be expressed as:

(3) $$i_{oq}=-\sum_{p=1,p\neq q}^{m}C_{p}\frac{du_{0}}{dt}+i_{R}+i_{L}$$

where *i_R_* and *i_L_* are the resistive and inductive components of *i_oq_*.

It can be found from (3) that if *i_R_* and *i_L_* are not considered, *i_oq_* and the sum of the zero-sequence current at the outlet of the non-faulty feeder is equal in magnitude, and the direction of change is opposite. Although *i_R_* and *i_L_* will affect the amplitude and phase of *i_oq_* to a certain extent, it can be found that there is a significant difference in the amplitude and dynamic change trend between the faulty feeder and the non-faulty feeder.

### 3.2. Principle of Faulty Feeder Identification Using Grey T-Type Correlation Degree

In order to express the similarity of the two curves, the traditional method of feeder identification based on similarity compares the static shapes of the two curves [39]. However, because the actual distribution network contains overhead lines and cables, and the capacitance to ground of the cables is much larger than the transmission lines, it leads to a large difference in the amplitude of the zero-sequence current. If the static correlation coefficient continues to be used, it will lead to misjudgment. In order to solve this problem, this article will use the grey T-type correlation degree. Based on the changing trend of the fault waveform, it can accurately indicate the similarity of the dynamic change trend of the non-faulted overhead line and cable, and no misjudgment of the line will be made.

Assuming that the transient signal sequence *S_k_* of the *k*^th^ feeder contains *n* data points in the sampling period, written as a row vector in the form *S_k_* = (*S_k1_*, *S_k2_*, …, *S_kn_*), then the transient signal composed of m feeders the matrix ***S*** is as follows:

(4) $$S=\left[\begin{array}{c} S_{1}\\ S_{2}\\ \ldots \\ S_{m} \end{array}\right]=\left[\begin{array}{cccc} S_{11} & S_{12} & \ldots & S_{1n}\\ S_{21} & S_{22} & \ldots & S_{2n}\\ \ldots & \ldots & \ldots & \ldots \\ S_{m1} & S_{m2} & \ldots & S_{mn} \end{array}\right]$$

The increment of the sequence *S_i_* at the $t_{0}^{th}$ sampling point can be expressed as

(5) $$\Delta S_{it_{0}}=S_{i(t_{0}+1)}-S_{i}{}_{t_{0}},t_{0}=1,2,\ldots ,n-1$$

The average value of the absolute value of the increment of the sequence in the sampling interval is:

(6) $$d_{i}=\frac{\sum_{t=1}^{n-1}\left|\Delta S_{it_{0}}\right|}{n-1}$$

The average value of the increment of the sequence *S_i_* at the $t_{0}^{th}$ sampling point is:

(7) $$Z_{it_{0}}=\frac{\Delta S_{it_{0}^{}}}{d_{i}}$$

The grey T-type correlation coefficient *ρ_ij_*[*S_it_*, *S_jt_*] of any two signal sequences *S_i_* and *S_j_* at the $t_{0}^{th}$ sampling point is defined as:

(8) $$\rho_{ij}\left[S_{it_{0}},S_{jt_{0}}\right]=\frac{\mathrm{sgn}(Z_{it_{0}},Z_{jt_{0}})}{1+0.5\left|\left|Z_{it_{0}}\right|-\left|Z_{jt_{0}}\right|\right|}$$

(9) $$\mathrm{sgn}(Z_{it_{0}},Z_{jt_{0}})=\{\begin{array}{c} 1,Z_{it_{0}}Z_{jt_{0}}\geq 0\\ -1,Z_{it_{0}}Z_{jt_{0}}<0 \end{array}$$

Then the grey T-type correlation coefficient *ρ_ij_* of *S_i_* and *S_j_* in the sampling period can be expressed as:

(10) $$\rho_{ij}=\frac{1}{n-1}\sum_{t_{0}=1}^{n-1}\rho_{ij}\left[S_{it_{0}},S_{jt_{0}}\right]$$

where *ρ_ij_*∈ [−1,1], the more similar the dynamic trends of *S_i_* and *S_j_* are, the closer *ρ_ij_* is to 1, otherwise it is closer to −1.

### 3.3. Identification Criterion Based on Grey T-Type Correlation Degree

According to the above method, the grey T-type correlation coefficient matrix ***ρ*** can be obtained:

(11) $$\rho =\left[\begin{array}{cccc} 1 & \rho_{12} & \ldots & \rho_{1m}\\ \rho_{21} & 1 & \ldots & \rho_{2m}\\ \ldots & \ldots & \ldots & \ldots \\ \rho_{m1} & \rho_{m2} & \ldots & 1 \end{array}\right]$$

The integrated grey T-type correlation coefficient of feeder *k* and other feeders can be defined as:

(12) $$\mu_{k}=\frac{1}{m-1}\sum_{j=1,j\neq i}^{m}\rho_{kj}$$

According to the above analysis, the line selection criteria can be determined as follows:

(13) $$\begin{array}{l} \mu_{k}=\min\left[\mu_{1},\mu_{2},\ldots ,\mu_{m}\right]\leq \mu_{set},\;\mathrm{the}\;k^{\mathrm{th}}\;\mathrm{feeder}\;\mathrm{fault}\\ \mu_{k}=\min\left[\mu_{1},\mu_{2},\ldots ,\mu_{m}\right]>\mu_{set},\;\mathrm{the}\;\mathrm{bus}\;\mathrm{fault}\; \end{array}$$

where $\mu_{set}$ is equal to 0.2 here.

**Figure 6:**
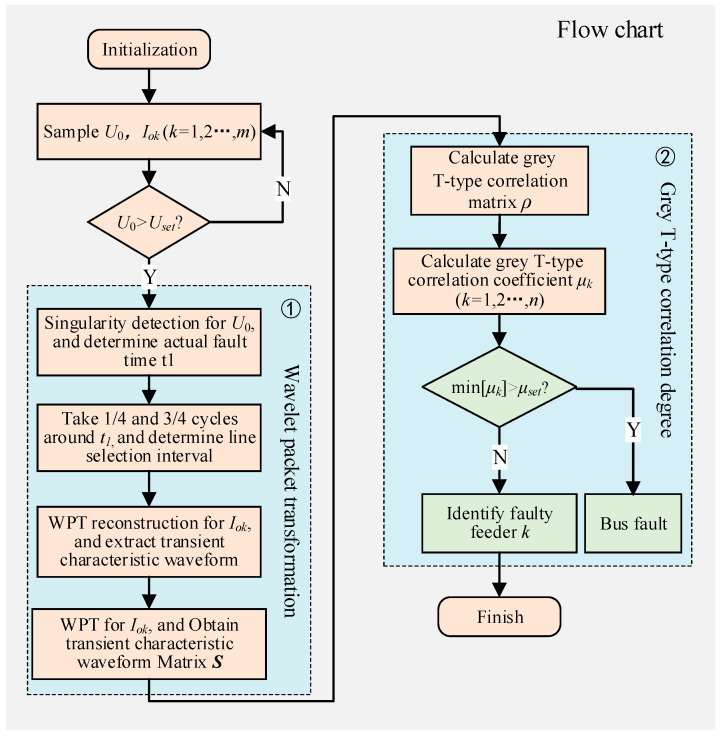
The flow chart of the proposed method.

Figure 6 is the flow chart of the proposed faulty feeder identification method combining wavelet packet and grey T-type correlation degree. Especially, in order to avoid misjudgment and distinguish bus faults (point of common connection of all feeders) from feeder faults, that is, to determine the bus fault as a feeder fault or the opposite condition, the threshold setting is necessary. A large number of simulation results show that the line selection accuracy is high when $\mu_{set}$ is set as 0.2. The threshold setting with too high or too low value will lead to confusion between feeder faults and bus faults. The steps can be described as follows: Firstly, by sampling the zero-sequence voltage/current and comparing them with thresholds, the ground fault is diagnosed; then, through wavelet packet transformation, the transient characteristic waveform is extracted, and the matrix S is obtained; after that the grey T-type correlation coefficient is calculated; finally, by analyzing $\mu_{set}$, the faulty feeder can be identified.

## 4. Verification Results and Discussion

**Figure 7:**
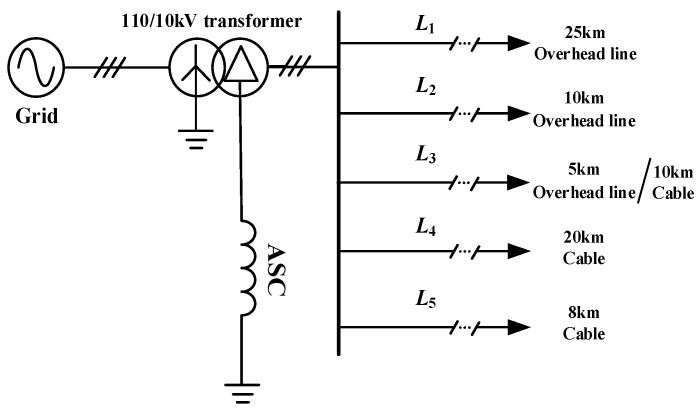
Schematic diagram of the simulation model: Two types of lines are considered.

As mentioned above, both the flexible grounding system and traditional resonant grounding system involve fault line selection. Due to its high controllability and excellent compensation performance, the former is attracting more and more attention. However, the flexible grounding system needs both fast and accurate line selection process. In order to verify the proposed faulty feeder identification method, the MATLAB/Simulink-based model is established, as shown in Figure 7. Especially, limited by the experimental conditions, and from the perspective of safety, it is a better choice to verify the proposed method by sufficient simulations. The electric distribution network model contains five feeders including two main types of lines: L1 is 25 km overhead line, L2 is 10 km overhead line, L3 is a hybrid line of 5 km overhead line and 10 km cable, L4 is 20 km cable, and L5 is 8 km Cable. The system voltage level is 110 kV, the rated voltage of the transformer is 110/10.5 kV, and the rated capacity is 50 MVA. The no-load current is 1%, the no-load loss is 35 kW, the short-circuit loss is 250 kW, and the short-circuit voltage ratio is 10%. The ground resistance is *R*_d_. The active load of each feeder is 4 × 10^6^ W, and the reactive load is 3 × 10^6^ Var. The line uses a distributed parameter model, and the parameter values are shown in Table 1. According to the mentioned parameters, the total ground capacity of the system ΣC = 1.26 × 10^−5^ F can be calculated and obtained. If the over-compensation degree of the arc suppression coil is 5%, the inductivity value of the arc suppression coil can be obtained:

(14) $$L=\frac{1}{1.05}*\frac{1}{3\omega^{2}\Sigma C}=0.243H$$

The internal resistance *R_L_* of the arc suppression coil is 10% of the inductive reactance, and *R_L_* = 8 Ω.

### 4.1. Characteristics of Transient Zero-Sequence Current

**Figure 8:**
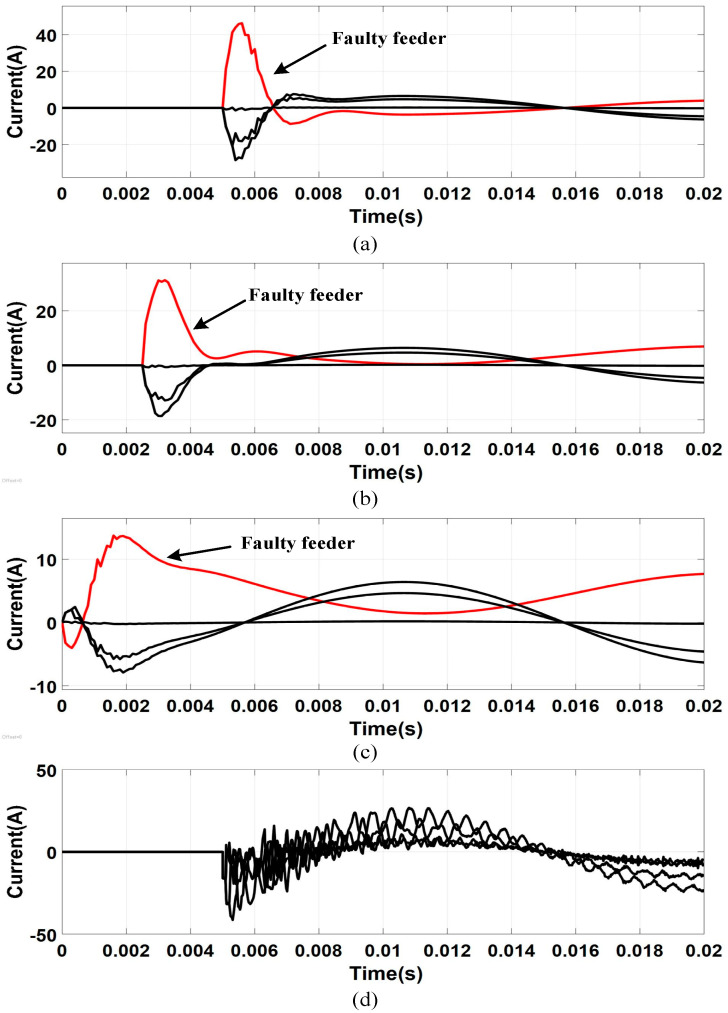
Transient zero-sequence current waveform: (**a**) Feeder fault, φ = π/2; (**b**) feeder fault, φ = π/4; (**c**) feeder fault, φ = 0; (**d**) bus fault, φ = π/2.

Figure 8 is the waveform diagram of the transient zero-sequence current during single-phase ground fault. It can be found that: (1) When the fault occurs on the feeder, no matter what time the fault occurs, the variation trend and amplitude of zero sequence current of the faulty feeder and normal feeder are very different; (2) the zero-sequence current of the normal feeders have a large difference in amplitude and a small difference in variation trend; (3) when the fault occurs on the bus, all the feeders can be regarded as normal feeders, the zero-sequence currents of all the feeders have a similar variation trend, and have a large difference in amplitude.

### 4.2. Faulty Feeder Identification under Typical Situations

The fault waveform is affected by the voltage phase angle *φ*, the grounding resistance *R_d_*, the fault location, and the type of fault line when the fault occurs. To verify the validity and rationality of the above line selection method under different influencing factors, three typical situations are considered in this article.

**Figure 9:**
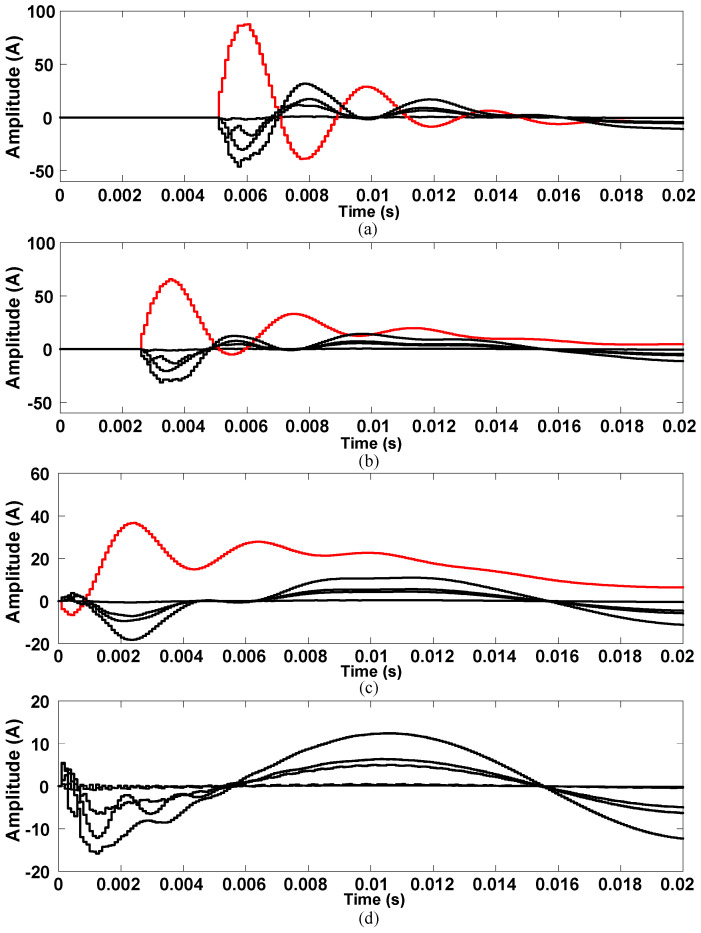
Transient waveforms when *R_d_* = 0 Ω: (**a**–**c**) Feeder faults; (**d**) bus fault.

**Figure 10:**
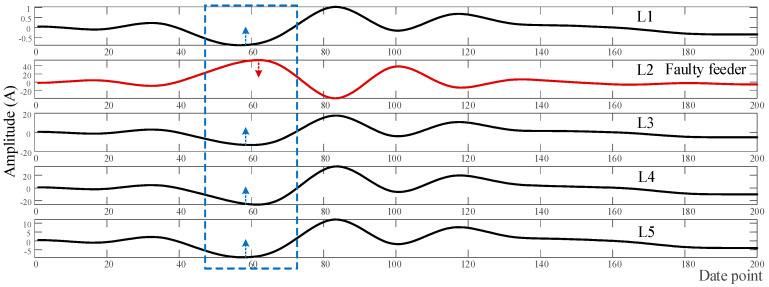
Transient characteristic waveforms extracted at (4, 0) node when *R_d_* = 0 Ω, *φ* = π/2.

Figure 9 and Figure 10 are the transient waveform of the zero-sequence current and the reconstructed waveform (*R_d_* = 0 Ω) at (4, 0) after extraction when the feeder fault occurs, respectively. The feeder faults and bus fault can be found in Figure 9a–d, respectively. In Figure 10, the *x*-axis represents the data points of the transient signal within the sampling period. It can be found that, compared with the original transient waveform, the extracted waveforms are filtered out of high-frequency interference. It smooths the waveform curve, strengthens the similarity between non-faulty feeders and the difference between faulty feeders and non-faulty feeders, and enhances the calculation accuracy of the grey T-type correlation selection coefficient.

The steps of judgment can be described as follows: Using the waveform after wavelet packet processing as the original waveform of the line selection; changing the fault occurrence line and location, respectively; calculating [*μ_1_, μ_2_, μ_3_, μ_4_, μ_5_*] according to the grey T-type correlation degree; and determining the faulty feeder according to (13), finally. Table 2 shows the results of line selection when *R_d_* = 0 Ω and *φ* = π/2. In this case, the transient process of the fault waveform is the largest, the characteristic quantity is the most obvious, and it is most conducive to the judgment of line selection; Table 3 shows the results of line selection when *R_d_* = 20 Ω and *φ* = π/4. In this case, the characteristic quantity is more obvious, which can represent the general resistance of low-resistance grounding of *φ*∈(0, π/2); Table 4 shows the results of line selection when *R_d_* = 2000 Ω and *φ* = 0. In this case, the high-resistance ground fault occurs. The fault transient process is the smallest, the feature quantity is the least obvious, the line selection is the most difficult, and the line selection accuracy is greatly disturbed. The accuracy of line selection under high-resistance ground fault is an important reference to evaluate the effectiveness of the line selection method.

Analyzing and comparing the above three typical situations, the following conclusions can be obtained: (1) No matter what type of fault, the correct rate of feeder identification is high, especially when a high resistance ground fault occurs; (2) In the non-faulty feeder, the correlation coefficient of the overhead line is not much different from that of the cable. It proves that the grey T-type correlation degree for feeder identification can effectively reflect the correlation of the dynamic change trend of overhead lines and cables, thereby reducing the misjudgment caused by the difference in the capacitance to ground between the cables and overhead lines; (3) the correlation coefficients between the faulty feeder and the non-faulty feeder are obviously different, and their change trend of the zero-sequence current is in the opposite direction; (4) bus fault can be regarded as a special case where all feeders are non-faulty feeders. In this case, the comprehensive grey T-type correlation coefficient of the zero-sequence current of each feeder is not much different.

## 5. Conclusions

In this article, based on the zero-sequence voltage/current sensor networks, a new faulty feeder identification method for the flexible grounding system combining grey T-type correlation degree and wavelet packet transform is proposed, which features both accuracy and rapidity. Different from the traditional method, this proposed method first extracts the transient characteristic waveform of the zero-sequence current of each feeder through the wavelet packet. In essence, it uses the timeliness of the wavelet packet to perform fast low-pass filtering on the signal to remove high-frequency interference and amplify the signal characteristics, which will promote accurate line selection. Then, through defining a comprehensive grey T-type correlation coefficient to represent the similarity between the dynamic change trends of the feeder, it can effectively reflect the correlation between the dynamic change trend of the overhead line and the cable, thereby reducing misjudgment due to the difference capacitance-to-ground value between the cable and overhead line. The proposed method can be applied in a flexible grounding system, which can meet the requirements of fast and accurate faulty feeder selection. Sufficient MATLAB-based simulation results verify the effectiveness and rationality of the proposed method and analysis.

Additionally, from the flow chart of the proposed method, it can be found that date sampling and calculation are the keys to the fault feeder identification. In the simulation model, the influence from the sampling time, missing data, and computation time can be ignored. However, when the proposed method is applied in practice, the above factors will affect the performance of faulty feeder identification to a certain extent. For example, the digital signal processor will determine the upper limit of the sampling frequency and the computation time. Besides that, due to the irrationality of the software program, the sampling data or processing data of intermediate links may be lost, which will influence the fault identification performance. In addition, when the saturation of the current transformer occurs, the current sampling error will become very large, which will affect the algorithm processing. These engineering problems need to be focused on in the future experimental stage.

## Figures and Tables

**Table 1:** Line parameters.

Types	Phase Sequence	R_0_ (Ω/km)	L_0_ (mH/km)	C_0_ (nF/km)
Overhead Lines	Positive sequence	0.132	1.258	9.780
	Zero sequence	0.389	4.126	7.758
Cable	Positive sequence	0.270	0.255	339
	Zero sequence	2.700	1.019	280

**Table 2:** The results of feeder identification when *R_d_* = 0 Ω and *φ* = π/2.

Fault Line	Fault Location/km	[*μ*_1_, *μ*_2_, *μ*_3_, *μ*_4,_ *μ*_5_]	Min[*μ*_1_, *μ*_2_, *μ*_3_, *μ*_4,_ *μ*_5_]	Line Selection Result
L_1_	10	[−0.5644, 0.5122, 0.4800, 0.4486, 0.5095]	−0.5644	L_1_
	25	[−0.5193, 0.4256, 0.4584, 0.4149, 0.4736]	−0.5193	L_1_
L_2_	5	[0.4241, −0.6755, 0.4115, 0.4367, 0.4846]	−0.6755	L_2_
	10	[0.5238, −0.5423, 0.5134, 0.4282, 0.5119]	−0.5423	L_2_
L_3_	5	[0.5963, 0.5845, −0.4122, 0.4638, 0.5735]	−0.4122	L_3_
	15	[0.6203, 0.6316, −0.2158, 0.5369, 0.6135]	−0.2158	L_3_
L_4_	10	[0.4839, 0.3656, 0.5552, 0.1218, 0.5569]	0.1218	L_4_
	20	[0.5763, 0.5554, 0.6210, −0.0796, 0.6216]	−0.0796	L_4_
L_5_	4	[0.5087, 0.4212, 0.5307, 0.4979, −0.0084]	−0.0084	L_5_
	8	[0.4731, 0.5204, 0.5399, 0.5347, −0.0585]	−0.0585	L_5_
Bus	0	[0.6351, 0.5808, 0.7460, 0.7594, 0.7642]	0.5808	Bus

**Table 3:** The results of feeder identification when *R_d_* = 20 Ω and *φ* = π/4.

Fault Line	Fault Location/km	[*μ*_1_, *μ*_2_, *μ*_3_, *μ*_4,_ *μ*_5_]	Min[*μ*_1_, *μ*_2_, *μ*_3_, *μ*_4,_ *μ*_5_]	Line Selection Result
L_1_	10	[−0.2235, 0.6375, 0.6312, 0.6296, 0.6438]	−0.2235	L_1_
	25	[−0.0702, 0.6839, 0.6558, 0.6459, 0.6381]	−0.0702	L_1_
L_2_	5	[0.6328, −0.0473, 0.6381, 0.6258, 0.6479]	−0.0473	L_2_
	10	[0.6996, −0.0059, 0.6036, 0.6184, 0.6328]	−0.0059	L_2_
L_3_	5	[0.6287, 0.6661, −0.0399, 0.5736, 0.6509]	−0.0399	L_3_
	15	[0.6689, 0.6586, −0.0403, 0.6254, 0.6564]	−0.0403	L_3_
L_4_	10	[0.6167, 0.5677, 0.6366, 0.0679, 0.6477]	0.0679	L_4_
	20	[0.5834, 0.5537, 0.6446, 0.0116, 0.6469]	0.0116	L_4_
L_5_	4	[0.5618, 0.5766, 0.5865, 0.6022, 0.0343]	0.0343	L_5_
	8	[0.5031, 0.5198, 0.5856, 0.5614, −0.0017]	−0.0017	L_5_
Bus	0	[0.7468, 0.7651, 0.8005, 0.8204, 0.8322]	0.7468	Bus

**Table 4:** The results of feeder identification when *R_d_* = 2000 Ω and *φ* = 0.

Fault Line	Fault Location/km	[*μ*_1_, *μ*_2_, *μ*_3_, *μ*_4,_ *μ*_5_]	Min[*μ*_1_, *μ*_2_, *μ*_3_, *μ*_4,_ *μ*_5_]	Line Selection Result
L_1_	10	[−0.6703, 0.4789, 0.4503, 0.4188, 0.4996]	−0.6703	L_1_
	25	[−0.7266, 0.4576, 0.4569, 0.4250, 0.4563]	−0.7266	L_1_
L_2_	5	[0.4239, −0.6702, 0.4241, 0.4264, 0.4461]	−0.6702	L_2_
	10	[0.2697, −0.6931, 0.3334, 0.3589, 0.3202]	−0.6931	L_2_
L_3_	5	[0.4817, 0.4935, −0.6572, 0.4517, 0.5066]	−0.6572	L_3_
	15	[0.5092, 0.4963, −0.6208, 0.4759, 0.5183]	−0.6208	L_3_
L_4_	10	[0.5100, 0.4516, 0.5161, −0.6454, 0.5204]	−0.6454	L_4_
	20	[0.5347, 0.5119, 0.5245, −0.6338, 0.5429]	−0.6338	L_4_
L_5_	4	[0.4646, 0.4326, 0.4692, 0.4588, −0.6833]	−0.6833	L_5_
	8	[0.4890, 0.4209, 0.4931, 0.4783, −0.6873]	−0.6873	L_5_
Bus	0	[0.7385, 0.8162, 0.7997, 0.7989, 0.7197]	0.7197	Bus

## Data Availability

The data presented in this study are available on request from the corresponding author.
